# MicroRNAs and lung cancer: overview of essential pathways and somatic mutations in cancer progression

**DOI:** 10.3389/fonc.2025.1677471

**Published:** 2025-10-07

**Authors:** Andrei-Alexandru Tirpe, Andreea Nutu, Constantin Busuioc, Ovidiu-Laurean Pop, Ioana Berindan-Neagoe

**Affiliations:** ^1^ Department of Genomics, MEDFUTURE Institute for Biomedical Research, Iuliu Haţieganu University of Medicine and Pharmacy, Cluj-Napoca, Romania; ^2^ Department of Pathology, National Institute of Infectious Disease, Bucharest, Romania; ^3^ Department of Pathology, Onco Team Diagnostic, Bucharest, Romania; ^4^ Department of Morphological Sciences, Faculty of Medicine and Pharmacy, University of Oradea, Oradea, Romania; ^5^ Doctoral School, Iuliu Hatieganu University of Medicine and Pharmacy, Cluj-Napoca, Romania; ^6^ The Academy of Medical Sciences, Bucharest, Romania

**Keywords:** microRNA, lung cancer, signaling cascade, targeted therapy, cancer progression, miRNA

## Abstract

Lung cancer is the most frequently diagnosed type of cancer worldwide, according to GLOBOCAN 2022 statistics. Key genetic alterations involve driver gene mutations that significantly enhance cancer aggressiveness. These include several *EGFR* mutations, *ALK* rearrangements, *ROS1* rearrangements, *RET* translocations, *MET* alterations, *NTRK* fusions, *BRAF* mutations and *KRAS* mutations, such as the *KRAS G12C* mutation. Naturally, each of these is part of a larger signaling pathway that becomes dysregulated via genetic alterations. We highlight the transduction of *EGFR: HER2* via *RAS-RAF-MEK-MAPK* pathway, *PI3K-PTEN-AKT* pathway and *STAT* pathway, of the *ALK* via *PI3K/AKT*, *MAPK/ERK* and *JAK/STAT* and of *KRAS* via effectors of the *MAPK* pathway and of the *PI3K* pathway. MicroRNAs (miRNAs) interfere at various levels with these pathways, either with pro-oncogenic effects or tumor suppressive effects. For instance, miR-33a is a tumor suppressive miRNA with a role in *EGFR*-tyrosine kinase inhibitor (TKI) resistance, miR-200c regulates the *ALK* pathway, and miR-22-3p regulates the *MET* pathway. The present paper also serves as an integrative work, highlighting the main cancer progression processes regulated by miRNAs, following these mutations. Specifically, we highlight the modulatory roles of miRNA in cancer cell survival and proliferation (miR-28, miR-30b/c), invasion and metastasis (miR-218, miR-182), neoangiogenesis (miR-29c), metabolic reprogramming (miR-124), and therapy resistance (miR-378, miR-328, miR-1244). The broad implications of miRNAs in lung cancer underline their potential real-world utility, as these entities can function as biomarkers for prognosis/diagnosis and even future therapeutic targets or agents.

## 1 Introduction

Lung cancer is an intricate malignancy, with an estimated 2.48 million new cases and 1.81 million deaths worldwide in 2022 alone (1). Many factors, including late diagnosis, patient characteristics and acquisition of cancer progression features can explain the poor prognosis (2). Comprehensive genomic characterization is increasingly recognized as essential in lung cancer management, as certain oncogene-driven lung cancers can benefit from targeted therapeutics. In specific cases, these targeted therapeutics can significantly prolong overall survival and/or progression-free survival compared to conventional chemotherapy and/or radiotherapy. For instance, in the PROFILE 1014 clinical trial, crizotinib, a first generation *ALK* tyrosine kinase inhibitor (TKI) significantly improved progression-free survival (PFS) in comparison to standard chemotherapy - median PFS (mPFS) = 10.9 months in the crizotinib group versus 7.0 months in the standard chemotherapy group; hazard ratio (HR) = 0.45, [95% CI 0.35-0.60], *p* < 0.001 for progression/death (3). Similarly, in patients with activating BRAF mutations, the combination of dabrafenib-trametinib in treatment-naive patients led to mPFS = 10.8 months [95% CI:7.0-14.5 months] and investigator-assessed median duration of response (mDoR) = 10.2 months [95% CI: 8.3-15.2 months] (4).

Histologically, lung cancer subtypes present significant differences that translate into prognostic differences, along with different treatment approaches. In general terms, lung cancers are classified as small cell lung cancers (SCLCs), which comprise about 15% of cases, and non-small cell lung cancers (NSCLCs), which comprise the rest of 85% of cases. NSCLC is further subclassified into lung adenocarcinoma (LUAD, with about 40% of total cases), lung squamous cell carcinoma (LUSC, 25-30%) and large cell carcinoma (LCC), with approximately 5-10% of all lung cancers (5, 6). Each of these lung cancer subtypes fosters diverse genomic and epigenomic alterations that develop and drive lung cancer from initiation to progression. For simplification purposes, the present paper will focus on NSCLC.

The human genome encompasses coding and non-coding regions, each playing distinct roles in cellular function (7). Coding regions, which make up about 2% of the genome, are responsible for producing proteins that perform various cellular functions. In contrast, non-coding regions, which constitute a large part of the genome, do not code for proteins. Instead, they are crucial in regulating gene expression and maintaining genomic stability (8). The interplay between the coding and non-coding regions is increasingly recognized as a fundamental aspect in the development and progression of various cancers, including lung cancer (9). Both the coding and non-coding genome are largely implicated in the carcinogenesis and progression processes of lung cancer. Specific genetic alterations in driver genes are the mainstay of targeted therapeutics in lung cancer, including subtypes such as LUAD. Some of the most well-known oncogenes and tumor suppressor genes associated with lung cancer include *EGFR*, *KRAS*, and *TP53* (10). These genetic alterations of the coding genome are pivotal in the initiation and progression of lung cancer, making them primary targets for therapeutic interventions. By comparison, the non-coding genome encompasses various elements, including promoters, enhancers, microRNAs (miRNAs), and long non-coding RNAs (lncRNAs), which interact with coding regions to influence cancer development and progression. Non-coding RNAs can modulate the epithelial-to-mesenchymal transition (EMT)/invasion, angiogenesis, and other progression processes (11). The functions and mechanisms of action are variable between different classes of ncRNAs, as is their biogenesis (12). MiRNAs are small non-coding RNAs of various lengths (approximately 22 nucleotides) generated via primary miRNAs (pri-miRNA), derived from introns or other non-coding transcripts (13). Their discovery by the Victor Ambros’ group (14) and Gary Ruvkun’s group (15, 16) revolutionized the field of non-coding RNAs. The main function of miRNAs includes mRNA degradation or translational repression via binding the 3’-UTR of a target mRNA through the miRNA-induced silencing complex (miRISC) (17). Other mechanisms of action include binding to coding regions and thus inhibiting protein expression, or binding to the 5’-UTR of mRNAs (12). Alternatively, miRNAs can also directly upregulate expression (18).

The present study serves as an integrative and comprehensive review of the current roles of non-coding RNAs in lung cancer progression processes, including neoangiogenesis, metastasis, immune evasion, and the acquisition of therapeutic resistance, with a specific focus on miRNAs. It also consolidates the main genetically altered genes that drive lung cancer. The novelty of this paper is given by the translational overview of miRNAs in NSCLC. The updated information regarding miRNAs implicated in this malignancy, in parallel with the main pathway alterations and driver mutations reinforces the approach.

## 2 The mutational landscape in lung cancer: genetic alterations, signaling pathways, and relevant miRNAs

NSCLCs present a significant number of alterations within their coding genome. For didactic purposes, this section will examine the NSCLC mutational landscape, focusing on genes that encode essential oncogenic drivers in specific subtypes of NSCLC, as well as miRNAs that are involved in modulating these entities. Specific genetic alterations render driver genes in NSCLC that enhance the proliferation rate of cancer cells and cancer aggressiveness. However, driver genes offer multiple therapeutic strategies by exploiting these elements as druggable targets. This section will briefly discuss the main driver genes and targetable mutations in NSCLC, along with relevant modulatory miRNAs and their potential applications.

### 2.1 Epidermal growth factor receptor

*EGFR* is part of the *HER/ErbB* family of receptor tyrosine kinases (RTKs) (19) and is found in approximately 15-40% of non-squamous NSCLC tumors (20); when activated, physiological non-mutated *EGFR* can recruit HER2 in heterodimers, forming the *EGFR::HER2* functional unit (21), leading to the downstream signaling pathways which promote cell survival, growth and migration (22). Intracellular signaling cascades pertain to the *RAS-RAF-MEK-MAPK* pathway, *PI3K-PTEN-AKT* pathway and *STAT* pathway (23), among others (22, 23). In lung cancer, the activated state is achieved through initial oncogenic mutations in exons 18-21, which encode the kinase domain (24). Two of the most frequent *EGFR* activating mutations are the *L858R* point mutation in exon 21 and the LREA in-frame deletion on exon 19 (19, 24). Activating *EGFR* mutations are druggable via *EGFR* TKIs, classified in first-generation *EGFR* TKIs, such as gefitinib and erlotinib and second-generation TKI afatinib (20). The exon 20 *T790M* “gatekeeper” mutation is considered one of the secondary *EGFR* point mutations in NSCLC. This mutation renders acquired resistance towards *EGFR* TKIs that do not specifically target this mutation (25). Third-generation *EGFR* TKI osimertinib is selective for the *T790M* resistance and for *EGFR*-TKI-sensitizing mutations. The FLAURA clinical trial showed that in patients with untreated *EGFR* mutation-positive (either *L858R* point mutation or exon 19 deletion) advanced NSCLC, the mPFS was significantly longer in patients treated with osimertinib than in the group treated with other standard *EGFR*-TKIs (gefitinib or erlotinib): 18.9 months versus 10.2 months, respectively, with HR = 0.46 [95% CI: 0.37-0.57, *p* < 0.001) for disease progression or death (26). This proves that osimertinib is superior to the other standard *EGFR*-TKIs. Further analysis in the FLAURA trial showed an improved overall survival (OS) of 38.6 months [95% CI: 34.5-41.8] in the group treated with osimertinib versus 31.8 months [95% CI: 26.6-36.0] in the group treated with other *EGFR*-TKIs, HR = 0.80 [95.05% CI: 0.64-1.00, *p* = 0.046] for death (27). Furthermore, recent clinical trial results show that in untreated *EGFR*-mutated (*L858R* mutation or exon 19 deletion) patients with advanced NSCLC, the addition of chemotherapy (pemetrexed + platinum-based agent in adjusted dosages) showed an increased investigator-assessed PFS, with HR = 0.62 [95% CI: 0.49-0.79, *p* < 0.001] for disease progression or death in comparison to the osimertinib monotherapy group. Moreover, 57% [95% CI: 50-63] of patients in the osimertinib-chemotherapy group were alive and disease progression-free at 24 months versus 41% [95% CI: 35-47] of patients receiving osimertinib monotherapy (28). The European Society for Medical Oncology Clinical Practice Guideline for Oncogene-Addicted Metastatic Non-Small-Cell Lung Cancer recommends the use of first-line *EGFR* TKIs in all patients with sensitizing *EGFR* mutation, regardless of performance status, gender, histology or tobacco exposure. In this case, osimertinib is the preferred first-line agent in patients with *L858R*/exon 21 or exon 19 deletions NSCLC and for patients with central nervous system metastases, as osimertinib can partly cross the blood-brain barrier (29).

Concomitantly, from a non-coding RNA perspective, there are several miRNAs associated with the modulation of *EGFR*-dependent processes in lung cancer. MiR-33a is involved in *EGFR*-TKI resistance. In gefitinib-resistant cells, resistance to TKIs is increased through *HDAC1*-dependent miR-33a suppression. This involves several processes, including cancer cell proliferation, migration, and apoptosis. Mechanistically, *HDAC1* bound *FOXK1* in gefitinib-resistant cells and silenced miR-33a. Conversely, miR-33a overexpression downregulated *ABCB7* and *p70S6K1* expression, leading to tumor-suppressive effects (30). Furthermore, miR-128b directly regulates *EGFR*. In tumor samples, loss of heterozygosity for miR-128b was frequent and correlated significantly with clinical response and survival after gefitinib treatment (31). In another study, let-7c expression led to increased cancer cell proliferation and invasion; high let-7c expression led to a reversal of EMT and increased cancer sell sensitivity to osimertinib via a *WNT1*- and *TCF-4*-dependent mechanism. From a more in-depth view, let-7c suppressed *WNT1* and *TCF-4* expression epigenetically via promoter methylation, leading to increased osimertinib activity upon the *EGFR*-mutated NSCLC cells (32).

### 2.2 *ALK* rearrangements

*ALK* rearrangement was first reported as a key driver in NSCLC by Soda et al. in 2007, when the authors identified the *EML4-ALK* fusion (33). Notably, other *ALK* fusions also exist (34). These alterations render constitutive *ALK* kinase activity (34). Usually, the *ALK* pathway includes downstream signaling via *PI3K/AKT*, *MAPK/ERK* and *JAK/STAT* pathways (35). Statistics indicate that rearrangements involving *ALK* are present in approximately 5% of NSCLCs (36, 37), while they are predominantly identified in adenocarcinomas (38). Tumors with *ALK* rearrangements exhibit aggressive behavior, including nodal metastasis and advanced stages at diagnosis (39). In this case, targeting these RTKs is a desideratum. First-line treatment options in NSCLC patients with ALK rearrangement include *ALK*-targeted TKIs such as crizotinib, alectinib, brigatinib and lorlatinib (29). Disease progression under treatment with crizotinib can be approached via newer-generation *ALK* TKIs, such as ceritinib (40) or alectinib (41), which have proven to have efficacy both intracranially and extracranially (40, 41). Furthermore, lorlatinib proved effective in subsequent lines of treatment in NSCLC patients previously treated with second-generation *ALK* TKIs, whilst presenting CNS activity (42).

In an *in vitro* and *in silico* study by Lai et al. on *EML4-ALK* mutant NSCLC cell lines, the authors found that miR-100-5p is a regulator of resistance to ALK TKIs. There was, however, no validation of the target. The *in silico* analysis identified the *mTOR* signaling pathway as a target (43). In a study by Fukuda et al., pretreatment with quisinostat, an *HDAC* inhibitor, resulted in the upregulation of miR-200c/141 promoter activity, thereby restoring miR-200c expression. This, in turn, reverted EMT. Next, administration of an *ALK* inhibitor can bypass EMT-induced therapy resistance (44). In a combined *in vitro* and *in vivo* study by Yun et al., treatment of cancer cells with panobinostat altered *H3K27ac* signal in promoters and enhancers, and led to activation of miRNAs with tumor suppressive effects, such as miR-449, followed by antiproliferative effects of ALK inhibitors upon resistant cancer cells, xenografts and *EML4-ALK* transgenic mice (45).

### 2.3 *ROS1* rearrangements

*ROS1* is a proto-oncogene that encodes a transmembrane protein with common structural features with *ALK* and insulin receptor families (46). *ROS1* protein-tyrosine kinase protein fusions are rare, occurring in an estimated 1-2% of NSCLC (47), predominantly in adenocarcinomas, but other histology types have also been described (48). Although the literature reports numerous fusion partners, CD74 is the most common (47). Other fusion partners include *SLC34A2, EZR and TMP3* (49). The *ROS1* fusions lead to constitutive *ROS1* kinase activity and increased cell survival, proliferation, and migration via the *STAT, PI3K, RAS/RAF/MEK/ERK1/2* and *Vav3* pathways (46). First-line treatment options in *ROS1* translocation metastatic NSCLC include crizotinib, entrectinib and repotrectinib as an alternative option (29). Second-line options for NSCLC patients who had systemic progression and received first-line ROS1 TKI include alternative next-generation *ROS1* TKI or platinum-based chemotherapy after rebiopsy (29).

One of the miRNAs that is in direct relation with *ROS1* is miR-760. In a study by Yan et al., miR-760 levels were found to be downregulated in 71.4% of NSCLC tissues considered and in NSCLC cell lines. In addition, overexpressing miR-760 led to inhibition of cancer cell proliferation, migration and cell cycle. Mechanistically, miR-760 inhibited *ROS1* expression in NSCLC cells and the miR-760 expression level was inversely correlated with *ROS1* expression level in NSCLC tissues (50, 51).

### 2.4 *RET* translocations

Another chromosomal rearrangement that drives NSCLC is the fusion of the rearranged during transfection (*RET*) gene with other fusion partners, such as *KIF5B* and *CCDC6*. This leads in turn to RET protein overexpression (52–54). Mechanistically, the *RET* gene encodes a proto-oncogene RTK which transduces to downstream *RAS/MAPK*, *PI3K/AKT* and *JNK* (53). *RET* rearrangements lead to fusion proteins that present a ligand-independent constitutive activation of *RET*, downstream pathway activation and stimulatory effects upon cancer cell growth, survival and proliferation (47). *RET* translocations are encountered in approximately 1-2% of NSCLC, predominantly adenocarcinomas (53) and predispose the patient to the development of brain metastases (55). Targeted therapeutics that act on RET fusions include selpercatinib and pralsetinib (29) and are recommended in patients previously untreated with RET inhibitors. Both drugs present high intracranial response rates (29).

### 2.5 MET

The *MET* tyrosine kinase is expressed on epithelial cells in various localizations. The *c-MET* proto-oncogene encodes MET and is a member of the RTKs family, with the main ligand being hepatocyte growth factor (HGF) (56). The activation of the *MET* TK activates the downstream signaling cascades *RAS/ERK/MAPK*, *PI3K/Akt*, *Wnt/β-catenin* and *STAT*. This modulates cell proliferation, survival and migration, among other processes (57). In general terms, the main *MET* pathological alterations include *MET* exon 14 skipping mutation (58) through various mutations – insertions, deletions, point mutations and others (59), *MET* amplification that is reported in up to 5% of NSCLC patients (60), *MET* overexpression, and the formation of *MET* fusion products. The fusion counterparts include *TPR, TRIM4 and HLA-DRB1* (59, 61). All these *MET* alterations lead to the activation of the MET tyrosine kinase with consecutive pro-oncogenic activity (56). In a clinical setting in NSCLC, capmatinib and tepotinib are FDA-approved for *MET* exon 14 skipping mutations but are not currently EMA-approved for first-line therapy (29). Capmatinib can also be used in patients with high *MET* amplification (>= 10 GCN) after immunotherapy and/or platinum-based chemotherapy, although this drug is currently not approved by the Food and Drug Administration (FDA) or European Medicines Agency (EMA) in this setting (29).

When considering a miRNA-dependent approach to lung cancers driven by *MET* alterations, several key points need to be addressed. First, in the Romano study, edited miR-411-5p (ed.miR-411-5p) induced *EGFR* TKIs sensitivity in gefitinib-resistant NSCLC cell lines that were only partially dependent on *MET* repression. Mechanistically, ed.miR-411-5p directly targeted *MET* and repressed the *MAPK* pathway (62). Moreover, in the Yang study, miR-22-3p was found to be downregulated in lung cancer tissues in comparison to normal lung tissues. MiR-22-3p mimics could reduce *MET* and *STAT3* expression, leading to the induction of apoptosis (63). In the Migliore study, epigenetically induced miR-205 expression in NSCLC cells resistant to MET-TKIs resulted in the downregulation of *ERRFI1*, leading to *EGFR* activation and sustained resistance to *MET*-TKIs. Conversely, the *in vivo* transduction of anti-miR-205 reversed crizotinib resistance.

Furthermore, in the absence of *EGFR* alterations, the *EGFR* activation via miR-205/ERRFI1 led to sensitivity of *MET*-TKI-resistant cells to combined *MET*-EGFR inhibition (64). MiR-182 was found to be downregulated in metastatic NSCLC cells in comparison to primary tumor tissues. Furthermore, overexpression of miR-182 inhibited cancer cell migration and invasion, reduced Snail expression and increased E-cadherin expression. Additionally, miR-182 directly suppressed Met expression. As such, the authors concluded that miR-182 may inhibit EMT and metastasis via inactivation of the *Met/AKT/Snail* pathway in NSCLC cells (65), highlighting the significant implications of these non-coding RNAs in specific lung cancer settings.

### 2.6 NTRK

The *NTRK* genes encode tropomyosin receptor kinases (TRKs) such as *TRKA*, *TRKB* and *TRKC* (66). NSCLC *NTRK* fusions have a variable prevalence, depending on the study (66); however, ESMO guidelines suggest a prevalence of less than 0.1% (29). *NTRK* fusions are obtained through intra- and interchromosomal rearrangements, as the 3’-sequence of *NTRK1*, *NTRK2* or *NTRK3* is fused with the 5’-sequences of various other genes (67). The chimeric product obtained by the fusion presents constitutive activation that is independent of ligands (68). This leads to activation of the downstream signaling pathways such as *MAPK*, *PI3K* and *PKC* (68). Targeted therapeutics, including larotrectinib and entrectinib, are recommended for patients with *NTRK* fusion-positive NSCLC who have no satisfactory alternative treatments (29).

### 2.7 *BRAF* mutations

In NSCLC, *BRAF* mutations occur in approximately 3-5% of cases and, for the most part, are mutually exclusive with *EGFR* mutations and *ALK* and *ROS1* rearrangements (29, 69). *BRAF* is a serine/threonine protein kinase of the RAF kinase family that can activate the *MAPK* signaling cascade via oncogenic mutations (70). Mechanistically, extracellular growth factors can activate RTK, activating the *SOS* family guanine nucleotide exchange factors (GEFs) and thus activating *RAS*. This cascade results in the activation and dimerization of *RAF* proteins via *GTP-RAS*, which further leads to the phosphorylation of *MEK1/2*, followed by the phosphorylation of *ERK1/2*. In its turn, *ERK1/2* phosphorylates downstream effectors, which modulate cell survival, proliferation, differentiation, and cell motility (71). The classification of *BRAF* mutations has led to a better understanding of the impact of these alterations. Class I BRAF mutations include *BRAF V600D/E/K/R* mutants that generate an important *BRAF* kinase activity, with a constitutive *MAPK* signaling activation. Class II and III *BRAF* mutations are *non-V600 BRAF* mutations. These are identified in the activation segment or P-loop for class II *BRAF* mutants, leading to *MAPK* pathway activation, and P-loop, catalytic loop, DFG motif for class III mutants, which present a lower basal kinase activity compared to wild-type *BRAF* or that do not present kinase activity (72). According to ESMO guidelines, patients who present with *BRAF* V600E mutations benefit from the dual therapy of dabrafenib in combination with trametinib in the setting of advanced or metastatic V600-mutated NSCLC (29). Of note is that the clinical trial only included patients with the *BRAF V600E* mutation (4).

### 2.8 *KRAS G12C* mutation

Activating *KRAS* mutations are observed in approximately 30% of NSCLC-LUAD cases, with the *KRAS G12C* mutation being the most common (73, 74). Oncogenic mutations in the *KRAS* gene are most prevalent at codons 12, 13 and 61 (75). These mutations activate *RAS* signaling through impairment of intrinsic GTPase or by alteration of *KRAS*, leading to the inability of *KRAS* to respond to GTPase-activating proteins (75). In general terms, *KRAS* mutations are associated with poor prognosis (76). The activation of *KRAS* leads to interaction with the effectors of the *MAPK* pathway – *RAF/MEK/ERK* – and with effectors of the *PI3K* pathway – *AKT/mTOR* –, among others (77–79), leading to cell survival and cell proliferation (80, 81). Current ESMO guidelines for NSCLC patients with *KRAS G12C* mutation recommend classic first-line treatment algorithms according to the non-oncogene addicted metastatic NSCLC (29, 82). Targeted therapeutics include sotorasib, which is recommended for patients with NSCLC with failed prior therapy, as the phase III clinical trial tested sotorasib in patients who progressed under platinum-based chemotherapy or ICI-based therapy (83). Another targeted therapeutic for the *KRAS G12C* mutation is adagrasib, which is currently approved only by the FDA and not by the EMA. Adagrasib was also studied in patients who progressed under platinum-based chemotherapy or ICI-based therapy and demonstrated an mPFS = 6.5 months [95% CI: 4.7-8.4] (84).

When considering the implications of miRNAs, several *KRAS*-related studies warrant mention. First and foremost, in the Xie study, miR-148a-3p inhibited NSCLC cancer cell proliferation and EMT by reducing *SOS2* expression, thereby decreasing *RAS* activation (85). In a study by Edmonds et al., miR-31 was found to be overexpressed in LUAD, overexpression that was independently correlated with reduced patient survival. In a transgenic mouse model, the induction of miR-31 led to lung hyperplasia, adenoma formation, and ultimately, the development of adenocarcinoma. In the Edmonds study, miR-31 promoted mutant *KRAS*-mediated oncogenesis by targeting and reducing the expression of negative regulators of *RAS/MAPK* cascade (86). In the Li study, *METTL3* promoted the m6A methylation of circ_0000620, leading to increased expression and stability, which in turn modulated the miR-216b-5p/KRAS axis and influenced apoptosis and cisplatin sensitivity in NSCLC cells.

Furthermore, transfection with si-circ_0000620 or miR-216b-5p mimic led to decreased *KRAS* expression in LUAD cells compared to the control group (87). In the Yan study, miR-1205 was found to directly bind 3’-UTR of *KRAS* and downregulate its expression. Furthermore, in a A549 xenograft model in nude mice, miR-1205 inhibited tumor growth and decreased levels of *KRAS*, *MDM4* and *E2F1* in tumor tissues (88, 173).

**Table 1** encompasses the targeted therapeutics discussed in this chapter.

**Table 1:** Targeted therapeutics in lung cancer, their aberrant targets and class/mechanisms.

Targeted therapeutic	Main aberrant target molecule/pathway indication	Class/Mechanism	References
Gefitinib	*EGFR*	First generation EGFR TKI	(29)
Erlotinib	*EGFR*	First generation EGFR TKI	(29)
Afatinib	*EGFR*	Second generation EGFR TKI	(29)
Osimertinib	*EGFR*	Third generation EGFR TKI; selective for exon 20 T790M gatekeeper mutation and EGFR-TKI-sensitizing mutations	(29)
Crizotinib	*ALK*	First generation ALK inhibitor; ROS1 inhibitor	(29, 89)
Alectinib	*ALK*	Second generation ALK inhibitor	(29, 89)
Brigatinib	*ALK*	Second generation ALK inhibitor; potent dual inhibitor for ALK L1196M and EGFR T790M mutations	(29, 89)
Lorlatinib	*ALK*	Third generation ALK and ROS1 inhibitor	(29, 89)
Ceritinib	*ALK*	Second generation ALK inhibitor	(29, 89)
Entrectinib	*ROS1*	TRKA/B/C, ROS1 and ALK TKI	(29, 90)
Repotrectinib	*ROS1*	Newer generation ROS1/TRK-ALK TKI	(29)
Selpercatinib	*RET*	Highly selective/potent RET inhibitor	(29, 91)
Pralsetinib	*RET*	Highly selective/potent RET inhibitor	(29, 91)
Capmatinib	*MET*	Selective MET TKI type Ib	(29, 56)
Tepotinib	*MET*	Selective MET TKI type Ib	(29, 56)
Dabrafenib/Trametinib	*BRAF*	BRAF inhibitor + MEK inhibitor for BRAF V600E mutation	(29, 92)
Sotorasib	*KRAS*	Direct KRAS G12C inhibitor	(29, 93)
Adagrasib	*KRAS*	Direct KRAS G12C inhibitor	(29, 93)

**Figure 1:**
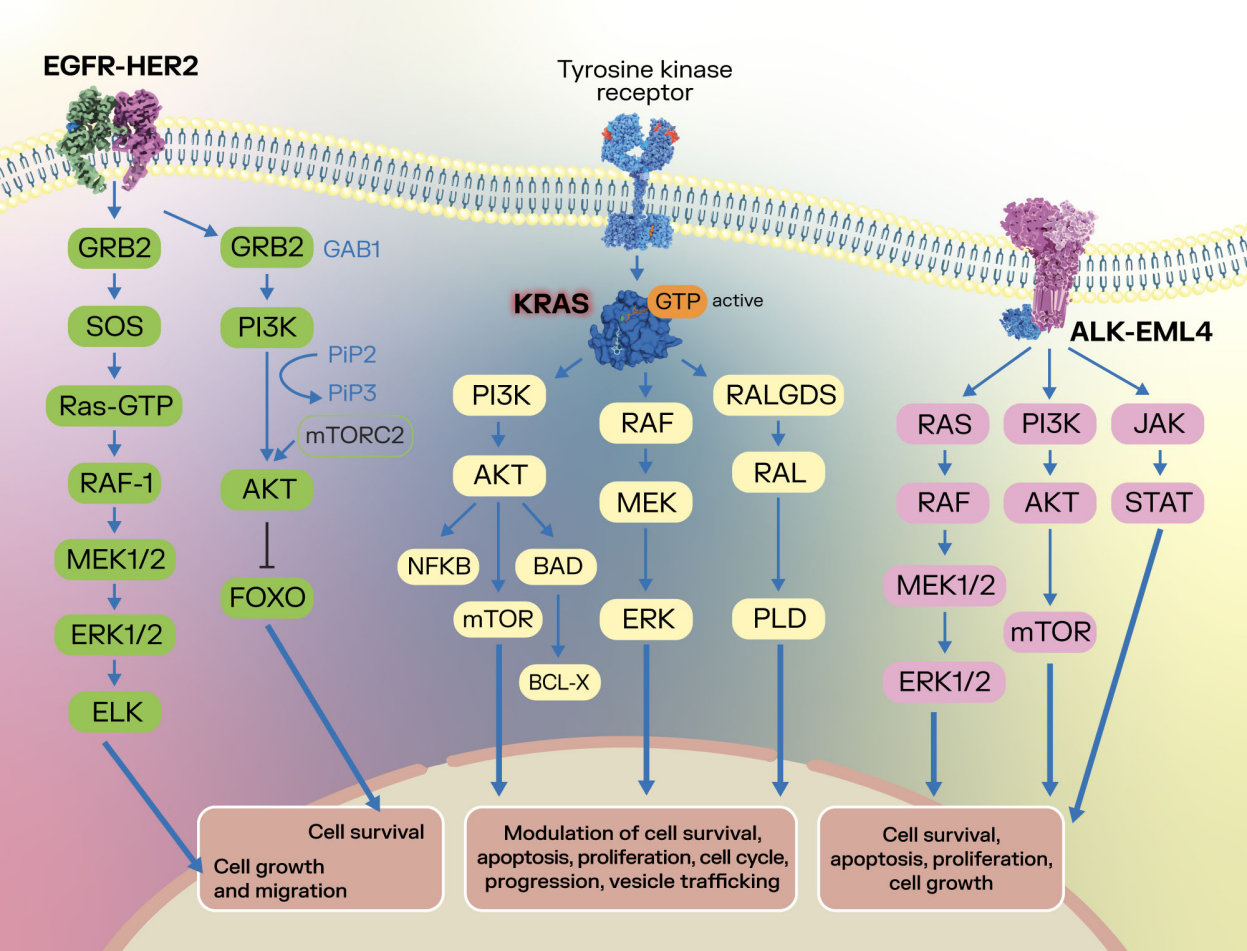
Summarization of several oncogenic targets and their signaling pathways in lung cancer. After binding with the corresponding ligands of the EGFR, the EGFR dimer activates downstream signaling pathways such as the RAS-RAF-MEK-ERK and PI3K-AKT, with an effect upon cell survival, cell growth and cell migration (19–21). In lung cancer, the activated state of EGFR is achieved through initial oncogenic mutations in exons 18-21, which encode the kinase domain (22). Concomitantly, KRAS plays a crucial role in signaling through the PI3K-AKT-mTOR, RAF-MEK-ERK and RALGDS-RAL-PLD pathways, modulating cell survival, apoptosis, cell proliferation, cell cycle, vesicle trafficking and progression (77–81), highlighting its oncogenic potential. In lung cancer, the ALK-EML4 fusion protein can form a dimer that does not require activation via ligand, which activates ALK and downstream RAS-RAF-MEK-ERK, PI3K-AKT-mTOR, and JAK-STAT signaling pathways, leading to effects upon cancer cell survival, apoptosis, proliferation and cell growth (33–35).

**Figure 1** summarizes some of the specific molecular targets discussed in the present chapter and their signaling pathways in lung cancer, highlighting their modulatory role in several cancer development processes.

Furthermore, we sought to provide an integrative perspective on the current understanding of the modulation of these key oncogenes involved in NSCLC via non-coding RNAs, specifically miRNAs. As entities that are widely implicated in carcinogenesis and cancer progression, miRNAs can exert their oncogenic or tumor suppressive capabilities through silencing of molecules taking part in the signaling cascades of these specific oncogenes. **Table 2** presents several key miRNAs that modulate specific target genes involved in these signaling cascades.

**Table 2:** A selection of miRNAs that target essential driver genes in lung cancer.

MiRNA	Effect	Expression level	Pathway affected	References
miR-33a	tumor suppressive	↓	EGFR	(30)
miR-128b	tumor suppressive	generally ↓	EGFR	(31)
let-7c	tumor suppressive	Not mentioned	EGFR	(32)
miR-100-5p	pro-oncogenic	↑	ALK/mTOR	(43)
miR-200c	tumor suppressive	↓	ALK	(44)
miR-449	tumor suppressive	↓	ALK	(45)
miR-760	tumor suppressive	↓	ROS1	(50, 51)
ed.miR-411-5p (A-to-I, pos. 5)	tumor suppressive	N/A	MET	(62)
miR-22-3p	tumor suppressive	↓	MET	(63)
miR-205	pro-oncogenic	↑	MET	(64)
miR-182	tumor suppressive	predominantly ↓	MET	(65)
miR-148a-3p	tumor suppressive	↓	Ras/MAPK/ERK	(85)
miR-31	pro-oncogenic	↑	Ras/MAPK	(86)
miR-216b-5p	tumor suppressive	↓/sponged by circ_0000620	KRAS	(87)
miR-1205	tumor suppressive	↓	KRAS	(88, 173)

Downregulated (↓), Upregulated (↑).

## 3 Dysregulation mechanisms of the non-coding genome in lung cancer

The non-coding genome describes the largest part of the human genome, as protein-coding sequences account for only approximately 1.5% of it (94). The non-coding genome is comprised of various entities, including non-coding regulatory regions such as non-coding RNAs (ncRNAs), promoters, enhancers and insulators, as well as untranslated regions (UTRs) (95). Advances in technology and high-throughput sequencing techniques led to the unravelment of this large part of the genome. As such, several types of ncRNAs have been described, some with a large number of evidence behind their proposed modulatory activity – miRNAs, long non-coding RNAs (lncRNAs), PIWI-interacting RNAs (piRNAs), small nuclear RNAs (snRNAs), small nucleolar RNAs (snoRNAs) and others (12). Although initially categorized as ncRNAs, recent reports suggest that some circRNAs may also have protein-coding abilities (96).

As miRNAs modulate numerous cellular processes, from cell growth and differentiation to development and apoptosis, it is abundantly clear that these entities play significant roles in malignancies (97). In cancer, miRNAs are categorized as oncogenes or tumor suppressors, depending on a given malignancy and the context (11); oncogenic miRNAs promote cancer traits and properties, while tumor suppressors have cancer suppressive abilities.

Regarding the practical interaction of miRNAs with the key updated hallmarks of cancer, as envisioned by Hanahan (98), it is comprehensible and realistic to expect the implication of modulatory miRNAs in each hallmark.

### 3.1 MiRNAs and the ECM

MiRNAs are significantly implicated in the regulation of the tumor microenvironment (TME), particularly in orchestrating and modulating the ECM. The dysregulated ECM has a significant role in lung cancer progression (99). Collagens are the most abundant proteins found within the ECM. Alteration in collagen expression influence TME cells (99). Discoidin domain receptor 1 (DDR1) is a RTK that binds collagen and activates downstream signaling cascades (100). DDR1 is largely implicated in cancer progression (101, 102). In a study by Ming et al., miR-199a-5p suppressed DDR1 expression. Circ_0087378 sponged miR-199a-5p and promoted malignant behavior through a DDR1-dependent mechanism (103). Concomitantly, integrins are proteins that intervene in the interaction between cells and ECM. This implies that integrins are involved in ECM remodeling and are largely implicated in cancer (104, 105). For instance, miR-338 may suppress lung cancer metastasis via integrin β3 (106). MiR-29c inhibits lung cancer metastasis and cancer cell adhesion to the ECM by integrin β1 and MMP2 expression inhibition (107). Moreover, exosomal miRNAs are involved in a variety of lung cancer processes, from cancer cell proliferation, apoptosis, epithelial-to-mesenchymal transition (EMT) and metastasis, to angiogenesis (108). Exosomes with low miR-34c-3p levels may promote NSCLC invasion and migration via upregulating integrin α2β1 (109).

### 3.2 MiRNAs and epigenetic regulation

MiRNAs are able to contribute to the epigenetic regulation via targeting several epigenetic regulators. MiR-101 interferes with lung cancer progression processes via the PTEN/AKT signaling cascade, by targeting the DNA-methyltransferase 3A (DNMT3A) (110). EZH2, a histone methyltransferase, functions as an epigenetic regulator by catalyzing the methylation of histone H3 - lysine 27 (111). In a study by Xia et al., anti-miR-21 led to a downregulation of EZH2 expression in lung cancer stem cells, proving the interaction between this miRNA and EZH2 (112). Another epigenetic modulators are histone deacetylases (HDACs), which interact with the chromatin structure and alter it, leading to transcriptional repression (113). In a study by Jeon et al., combined treatment with miR-449a and HDAC inhibitors *in vitro* led to a significant reduction in growth when compared to treatment with HDAC inhibitors alone (114). MiR-200b is involved in the therapeutic resistance of LUAD cells via E2F3; the suppression of HDAC1/4 increases miR-200b expression via histone-H3 acetylation upregulation, suggesting a crosstalk between miR-200b and HDAC1/4. In the Chen study, HDAC1/4 silencing led to G2/M cell cycle arrest, inhibited cancer cell proliferative abilities, increased cancer cell apoptosis, and counteracted the therapeutic resistance in docetaxel-resistant LUAD cells, in part via a miR-200b-controlled mechanism (115).

**Figure 2:**
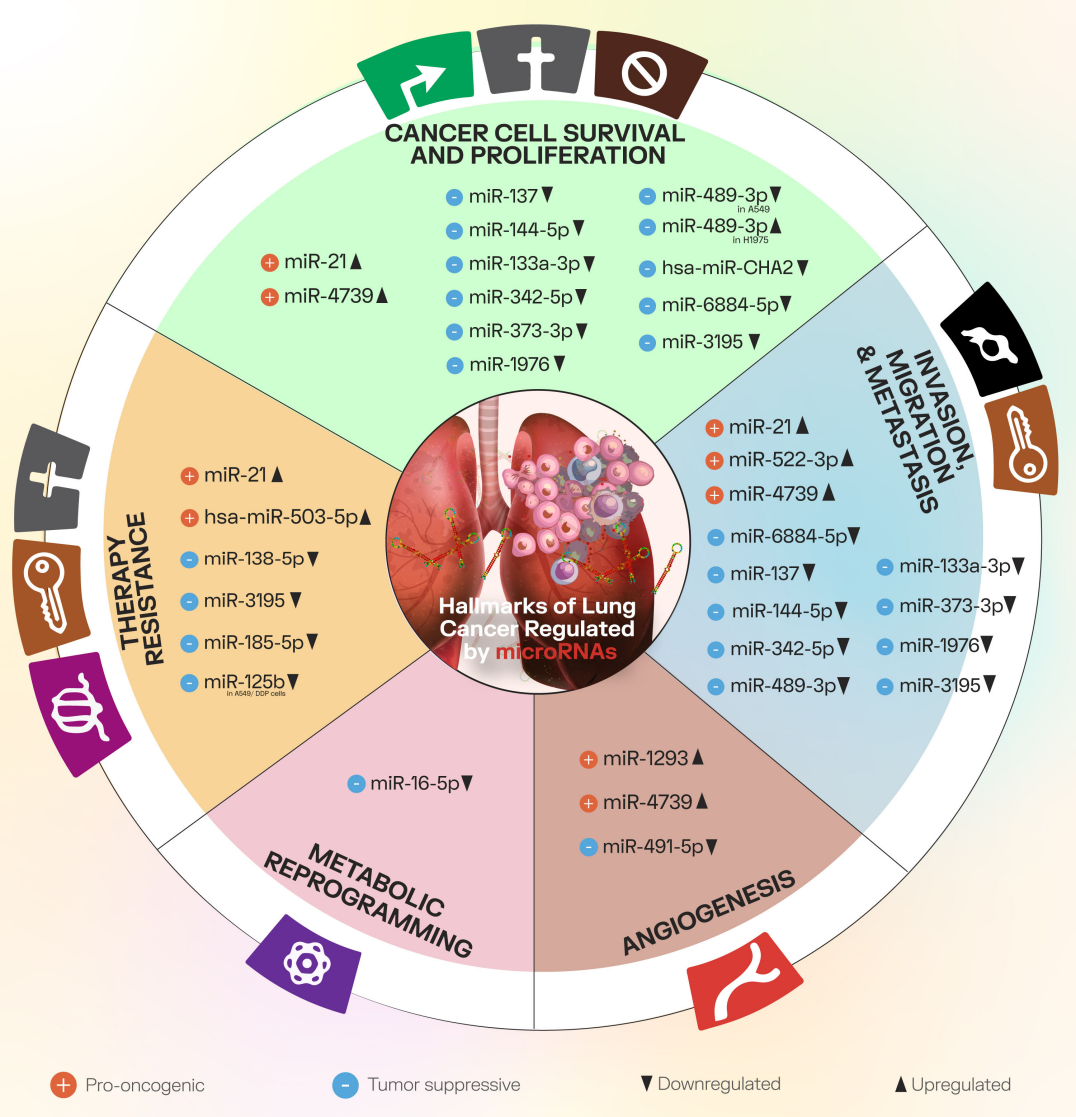
The main cancer progression processes discussed in the present paper, along with downregulated and upregulated miRNAs that modulate factors involved in these processes.

Furthermore, in the present chapter, we will briefly discuss some of the main NSCLC progression processes, providing several practical examples of modulatory miRNAs from the literature. **Figure 2** presents these cancer processes and the associated miRNAs.

### 3.3 Survival and proliferation

MiRNAs can modulate NSCLC cell survival and proliferation. In a study by Gong et al., miR-20a promoted NSCLC cell proliferation through *PTEN* inhibition and upregulation of PD-L1 (116). In another study by Cui et al., the expression of miR-28 was significantly upregulated in NSCLC tissues and cell lines compared to adjacent normal tissue and control cell lines, respectively. Furthermore, miR-28 promoted cancer cell proliferation by directly targeting *PTEN* (117). When referring to miR-30b/c, Zhong et al. found that miR-30b/c levels were downregulated in NSCLC specimens in comparison to control – adjacent non-tumor tissues and that miR-30b/c targeted *Rab18* in a direct manner leading to a downregulation in *Rab18* expression and inhibition of NSCLC cell proliferation (118). Moreover, Peng et al. identified miR-19 as an oncogenic miRNA that is overexpressed in NSCLC tissues and several lung cancer cell lines. Herein, miR-19 inhibited *CBX7* expression via CBX7 3’-UTR binding, leading to a decrease in *CBX7* mRNA expression and *CBX7* protein levels. Overexpression of miR-19 may significantly improve NSCLC cell proliferation and migration (119). Conversely, Liu et al. showed that miR-1253 was significantly downregulated in NSCLC tissues and associated with several clinical parameters such as advanced clinical stage, lymph node metastasis and even poor survival, highlighting the relevance of miRNAs in this malignancy. In the Liu study, overexpression of miR-1253 inhibited cancer cell proliferation, migration and invasion *in vitro* and identified the long isoform of *WNT5A* as a target for miR-1253 (120). Lastly, a study by Yoo et al. found that miR-CHA1 expression was downregulated in human lung cancer tissues (LUAD, SqCC) and cell lines. Overexpression of miR-CHA1 reduced *XIAP* mRNA expression and protein levels and thus inhibited NSCLC cell proliferation and induced apoptosis both *in vitro* and *in vivo* (121).

### 3.4 Invasion and metastasis

As versatile modulators of cancer progression processes, miRNAs control invasion and metastasis, among other factors from the local TME, such as hypoxia (122). In general terms, metastasis implies in its first phase the epithelial-to-mesenchymal transition, with consecutive loss of epithelial-like characteristics of cancer cells to a mesenchymal-like state. This leads to increased motility, invasiveness and the ability to degrade the ECM (123). Following EMT, cancer cells may regress back towards epithelial-like characteristics via mesenchymal-epithelial transition (MET) (123). In a study by Shi et al., miR-218 expression was found to be significantly downregulated in lung cancer tissues in comparison to control; the authors identified an association between miR-218 levels and lymph node metastasis and histological grade (124). Mechanistically, miR-218 suppressed invasion and metastatic spread via targeting *Slug* and *ZEB2*, key factors in the EMT process, and increased chemosensitivity to cisplatin in H1299 via *Slug* and *ZEB2* (124). Moreover, Li et al. found that miR-182 is downregulated in metastatic NSCLC cells in comparison to primary tumor tissues; miR-182 overexpression reduced *Snail* expression and conversely promoted E-cadherin expression, leading to the inhibition of migration and invasion in lung cancer cells. In addition, miR-182 silenced *MET* expression, inhibited *AKT* phosphorylation and nuclear *Snail* accumulation. Li et al. concluded that miR-182 may inhibit NSCLC metastasis and EMT through the inactivation of *MET/AKT/Snail* signaling (65). Conversely, miRNAs can be regulated by a diverse number of factors. In the Chang et al. study, the authors found that miR-137 expression is induced by *Slug* and promoted metastasis via targeting transcription factor *AP-2 gamma (TFAP2C)* (125). MiR-137 knockdown inhibited *Slug*-induced NSCLC cell invasion and migration (125).

### 3.5 Neoangiogenesis

NSCLC progression is dependent on generating new vascularization via the neoangiogenic process, sustaining the relative oxygenation inside the TME. Our group has critically reviewed the neoangiogenesis process in NSCLC and the implication of miRNAs as potential neoangiogenesis-related therapeutic agents/targets in another paper (126). In general terms, neoangiogenesis is governed by pro-angiogenic factors, including *VEGFA*, *FGF2*, *PDGFB*, *EGF*, *MMP2*, and anti-angiogenic factors, such as *THBS1* and *TIMP1*. Neoangiogenesis is driven by the imbalance between the pro-angiogenic and anti-angiogenic factors, termed the angiogenic switch (126). Intuitively, the miRNA-related modulation of neoangiogenesis can be achieved via miRNAs that target either pro-angiogenic or anti-angiogenic factors. In a lung cancer study by Liu et al., miR-29c was found to target VEGFA and act as a tumor suppressor by inhibiting cancer cell proliferation, migration and invasion, and angiogenesis *in vitro*. Furthermore, the authors identified a significant association between the downregulated miR-29c expression and poor prognosis in LUAD stage IIIA-N2 patients (127). In another study by Mao et al., tumor-derived miR-494 targeted and downregulated *PTEN* with a consecutive activation of the *AKT/eNOS* signaling pathway within human vascular endothelial cells, thus promoting neoangiogenesis (128).

### 3.6 Metabolic reprogramming

Cancer cells are able to reprogram their metabolism in order to increase the uptake of nutrients and promote their survival, growth and proliferation (129–131). One such alteration is the Warburg effect – a metabolism switch towards the use of glycolysis even in aerobic conditions (130, 131). Naturally, miRNAs can regulate factors that are in relation with metabolic rewiring as well. For instance, miR-124 overexpression inhibited NSCLC cell growth, energy metabolism, glucose consumption and lactate production via targeting glucose transporter 1 (*GLUT1*) and hexokinase II (*HKII*) and negatively regulating *AKT1* and *AKT2* (132).

### 3.7 Therapy resistance

Therapy resistance is an essential process in the evolution of NSCLC under treatment and represents a major challenge for clinicians, as it leads to therapeutic failure and cancer progression. In non-oncogene-addicted metastatic NSCLC, platinum-based chemotherapeutics remain a viable treatment option in various circumstances (82). Therapy resistance to cisplatin (cDDP) is multifaceted, as multiple mechanisms might be involved at a certain point. Such mechanisms include efflux transporters, EMT, autophagy, and modulation of different signaling cascades that pertain to cancer cell survival and apoptosis (133). For instance, miR-378 upregulation in cell lines A549/cDDP and Anip973/cDDP led to secreted form clusterin (sCLU) expression downregulation via direct targeting, sensitizing the NSCLC cells to cDDP. Concomitantly, patients sensitive to cDDP had higher levels of miR-378 and lower levels of sCLU in tumor tissues (134). In a study by Wang et al., miR-328 expression was significantly increased, and *PTEN* mRNA expression level was significantly decreased in tumor tissues from cDDP-resistant NSCLC patients in comparison to cDDP-sensitive NSCLC patients. Furthermore, the authors observed a higher miR-328 expression level and lower *PTEN* expression level in the cDDP-resistant A549 cell line (A549rCDDP) compared to the parental A549 cell line and confirmed that miR-328 targeted PTEN. Wang et al. showed that the inhibition of miR-328 in A549rCDDP cells treated with cDDP induced apoptosis and decreased cancer cell proliferation, highlighting the association of miR-328/PTEN in NSCLC cDDP resistance (135). In another study by Li et al., miR-589 and miR-1244 were downregulated in A549/cDDP cells in comparison to the parental A549 cell line, while the expression of miR-182 and miR-224 was found to be increased in the A549/cDDP cell line, with statistical significance. Transfection of the resistant A549/cDDP cells with miRNA mimics miR-589 or miR-1244 led to an improvement in cDDP sensitivity, reducing cancer cell invasion and apoptosis and underlining the implication of these miRNAs in cDDP chemosensitivity (136).

**Figure 3:**
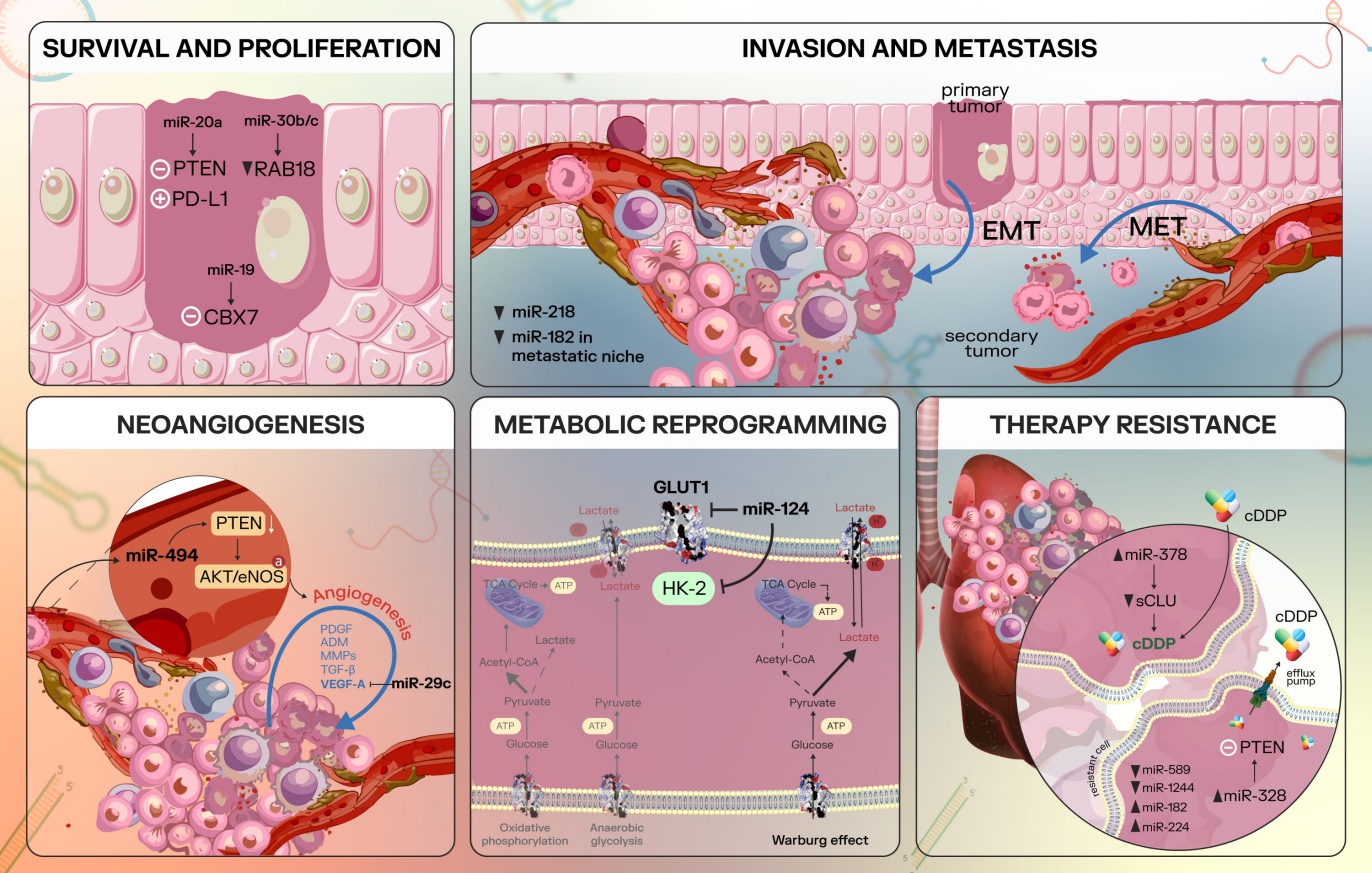
Overview of some cancer processes regulated by microRNAs in lung cancer. Survival and Proliferation: in lung cancer, miR-20a inhibits PTEN and upregulates PD-L1, promoting NSCLC cell proliferation (116); miR-30b/c is able to target Rab18 directly, leading to Rab18 repression and inhibition of NSCLC cell proliferation (118); miR-19 is an oncogenic miRNA that inhibits CBX7 expression (119). Invasion and metastasis: metastasis implies several essential processes, including epithelial-to-mesenchymal transition, with increased cancer cell invasiveness, motility and the ability to degrade the ECM. Furthermore, the mesenchymal-to-epithelial transition reverses these mesenchymal characteristics to epithelial-like characteristics (122, 123). In lung cancer, miR-218 is able to suppress invasion and metastatic spread by targeting key factors that modulate the EMT process - Slug and ZEB2 (124). In addition, a study found decreased miR-182 levels in metastatic NSCLC cells compared to the primary tumor and miR-182 may inhibit NSCLC EMT via inactivation of MET/AKT/Snail signaling pathway (65). Neoangiogenesis: essential pro-angiogenic factors include VEGFA, FGF2, PDGFB, EGF, MMP2 (126); miR-29c is able to target VEGFA and thus act as a tumor suppressor (127). MiR-494 targets and downregulates PTEN, with a consecutive activation of the AKT/eNOS pathway and stimulation of neoangiogenesis (128). Metabolic reprogramming: miR-124 interferes with cancer cell metabolism by targeting GLUT1 and HKII, negatively regulating AKT1 and AKT2 (132). Therapy resistance: miRNAs are largely implicated in therapy resistance in NSCLC, which is a multifaceted process. MiR-378 upregulation downregulates secreted form clusterin (sCLU) via direct targeting and led to sensitizing the NSCLC cells to cDDP (134). Other miRNAs implicated in NSCLC therapy resistance include the miR-328/PTEN duo (135), miR-589, miR-1244, miR-182, and miR-224 (136), with various expression levels of these non-coding RNAs.

**Figure 3** provides a visual representation of the implication of miRNAs in specific lung cancer processes.

Furthermore, **Table 3** integrates a larger variety of miRNAs that modulate essential cancer processes, exemplifying their wide distribution in this malignancy.

**Table 3:** A selection of miRNAs that modulate the main cancer processes.

Cancer process	MiRNA	Expression level	Study type	Stimulatory (+) Inhibitory (-)	Proposed effect	Ref
Cancer cell survival and proliferation	miR-21	↑	Review	(+) proliferation	Pro-oncogenic	(137)
	miR-4739	↑	*in vitro, in vivo, ex vivo (tissues)*	(+) proliferation	Pro-oncogenic	(138)
	miR-6884-5p	↓	*in vitro*	(–) proliferation	Tumor suppressive	(139)
	miR-144-5p	↓	*in vitro*	(-) proliferation	Tumor suppressive	(140)
	miR-200c-3p	–	*in vitro*	(-) proliferation	Tumor suppressive	(141)
	miR-342-5p	↓	*in vitro, in vivo, ex vivo (tissues)*	(-) proliferation	Tumor suppressive	(142)
	miR-489-3p	↓ in A549 ↑ in H1975	*in vitro*	(-) proliferation	Tumor suppressive	(143)
	miR-137	↓	*in vitro, in vivo, ex vivo (tissues), in silico*	(-) proliferation	Tumor suppressive	(144)
	miR-3195	↓	*in vitro, in vivo*	(-) proliferation	Tumor suppressive	(145)
	hsa-miR-CHA2	↓	*in vitro, in vivo, ex vivo (tissues)*	(-) proliferation	Tumor suppressive	(146)
	miR-1976	↓	*in vitro, in vivo, ex vivo (tissues)*	(-) proliferation	Tumor suppressive	(147)
	miR-373-3p	↓	*in vitro, ex vivo (tissues)*	(-) proliferation	Tumor suppressive	(148)
	miR-133a-3p	↓	*in vitro, in vivo*	(-) proliferation	Tumor suppressive	(149)
Invasion, migration and metastasis	miR-21	↑	Review	(+) metastasis, mesenchymal-epithelial transition	Pro-oncogenic	(137)
	miR-522-3p	↑	*in vitro, in vivo, ex vivo (tissues)*	(+) metastasis	Pro-oncogenic	(150)
	miR-4739	↑	*in vitro, in vivo, ex vivo (tissues)*	(+) migration, metastasis	Pro-oncogenic	(138)
	miR-4448	–	*in vitro, ex vivo*	(-) EMT	Tumor suppressive *SCLC	(151)
	miR-6884-5p	↓	*in vitro*	(-) EMT	Tumor suppressive	(139)
	miR-144-5p	↓	*in vitro*	(-) invasion, migration	Tumor suppressive	(140)
	miR-200c-3p	–	*in vitro*	(-) invasion	Tumor suppressive	(141)
	miR-342-5p	↓	*in vitro, in vivo, ex vivo (tissues)*	(-) invasion, migration	Tumor suppressive	(142)
	miR-489-3p	↓	*in vitro*	(-) migration	Tumor suppressive	(143)
	miR-137	↓	*in vitro, in vivo, ex vivo (tissues), in silico*	(-) migration, invasion	Tumor suppressive	(144)
	miR-3195	↓	*in vitro, in vivo*	(-) migration	Tumor suppressive	(145)
	miR-1976	↓	*in vitro, in vivo, ex vivo (tissues)*	(-) migration	Tumor suppressive	(147)
	miR-373-3p	↓	*in vitro, ex vivo (tissues)*	(-) invasion, migration	Tumor suppressive	(148)
	miR-133a-3p	↓	*in vitro, in vivo*	(-) metastasis	Tumor suppressive	(149)
Neoangiogenesis	miR-1293	↑	*in vitro, in vivo*	(+) angiogenesis	Pro-oncogenic	(152)
	miR-4739	↑	*in vitro, in vivo, ex vivo (tissues)*	(+) angiogenesis	Pro-oncogenic	(138)
	miR-491-5p	↓	*in vitro, in vivo*	(-) angiogenesis	Tumor suppressive	(153)
Metabolic reprogramming	miR-183-5p	–	*in vitro, in vivo*	(+) mitoROS production	Tumor suppressive	(154)
	miR-16-5p	↓	*in vitro, ex vivo (tissues)*	(-) lactate accumulation (-) glucose uptake (-) ATP levels	Tumor suppressive	(155)
Therapy resistance	miR-21	↑	Review	(+) therapy resistance	Pro-oncogenic	(137)
	hsa-miR-503-5p	↑	*in vitro, in vivo*	(+) therapy resistance	Pro-oncogenic	(156)
	miR-138-5p	↓ in gefitinib-resistant cells	*in vitro, in vivo*	(-) therapy resistance	Tumor suppressive	(157)
	miR-3195	↓	*in vitro, in vivo*	(-) therapy resistance	Tumor suppressive	(145)
	miR-185-5p	↓ in drug resistance	*in vitro, in silico*	(+) chemosensitivity	Tumor suppressive	(158)
	miR-125b	↓ in both A549 and A549/DDP cells	*in vitro, in vivo, ex vivo*	(-) therapy resistance	Tumor suppressive	(159)

Downregulated (downwards arrow), Upregulated (upwards arrow).

## 4 Implications and future directions

As exemplified, lung cancer is an intricate malignancy with high incidence and poor overall prognosis. Recent developments in the field have brought a modest improvement in patient survival, with the addition of immunotherapy and targeted therapeutics in specific circumstances. Herein, we have critically reviewed the main driver mutations that may be identified in lung cancer, along with essential data regarding the associated signaling pathways and their modulation via non-coding RNAs, specifically miRNAs.

MiRNAs emerged as key players with modulatory roles across the spectra of cancer processes - from lung cancer development, to progression. Indeed, these entities are able to modulate cancer cell proliferation, invasion and metastasis, neoangiogenesis, metabolic effects, therapy resistance and other. Hence, considering their large implication in cancer, the interest in their use as therapeutic targets, agents, or even biomarkers for diagnosis/prognosis is increasing.

From a biomarker perspective, in a study by Liu et al. (160), the authors analyzed in 168 early-stage NSCLC patients, 100 healthy volunteers and 128 patients with benign lung nodules a number of clinical and biochemical parameters, along with miR-200 expression in peripheral blood-derived extracellular vesicles (EVs). The parameters taken into account included carbohydrate antigen 199 (CA199), carbohydrate antigen 242 (CA242), carcinoembryonic antigen (CEA), interleukin-6 (IL-6) and tumor necrosis factor-α (TNF-α). Interestingly, peripheral blood-derived miR-200 EVs displayed diagnostic value, with a sensitivity of 60.12%, specificity of 95.18%, area under the curve (AUC) = 0.855 [95% CI: 0.816-0.888], p < 0.001 for early-stage NSCLC. Furthermore, the addition of CA242, CEA and CA199 besides peripheral blood-derived miR-200 EVs displayed increased diagnostic efficacy, with a sensitivity of 89.88%, specificity of 98.68% and AUC = 0.942 [95% CI: 0.914-0.964], p < 0.001 for early-stage NSCLC, rendering the potential utility of miR-200 as a biomarker in this malignancy (160).

Furthermore, Wozniak et al. (161) sampled 100 NSCLC patients stages I to IIIA, marked as early-stage NSCLC and 100 non-cancer controls, screening 754 circulating miRNAs through qRT-PCR. This led to a model of 24-miRNA panel which can be consulted in the Wozniak study (161). Herein, logistic regression analyses showed diagnostic potential for the 24-miRNA panel with an AUC = 0.92 [95% CI: 0.87-0.95] in discriminating lung cancer cases from controls. Furthermore, when adjusted to sex, age and smoking status, the diagnostic efficacy of the combined 24-miRNA panel increased to an AUC = 0.94 [95% CI: 0.90-0.97]. Moreover, the 24-miRNA panel had similar performances across the different NSCLC subtypes - with an AUC = 0.94 for LUADs and AUC = 0.96 for LUSCs. In addition, subgroup analyses showed AUC = 0.96 for stage I (IA and IB), AUC = 0.98 for stage II (IIA and IIB) and AUC = 0.97 for stage IIIA NSCLC patients (161).

In a study by Wang et al. (162), the authors overlapped miRNA data set from miRNA sequencing data of 8 specimens that were collected from thoracic surgery of non-smoking female patients with LUAD and data extracted from the TCGA database. The authors identified hsa-miR-200a, hsa-miR-21 and hsa-miR-584 as miRNAs significantly associated with OS in this subset of patients, highlighting the potential of these miRNAs to be used as a prognostic model (162).

Abdipourbozorgbaghi et al. (163) analyzed plasma miRNA expression in a cohort of 122 patients - 78 NSCLC patients and 44 healthy controls. Although the authors found that miRNA expression levels in LUAD were independent of tumor stage, 2 miRNAs were identified as early-stage (stage I and II) biomarkers - hsa-miR-210-3p and hsa-miR-301a-5p, whilst hsa-miR-9-5p, hsa-miR-141-5p and hsa-miR-147b-3p were identified as late-stage (stage III and IV) biomarkers. Conversely, there was a higher variability between the stages, with only 6 miRNAs being common. Hsa-miR-210-3p and hsa-miR-301a-5p were found to be early-stage miRNAs in both LUAD and LUSC, and hsa-miR-9-5p was a late-stage biomarker in both LUAD and LUSC patients. The authors concluded with one miRNA diagnosis panel that included 7 miRNAs for LUAD diagnosis and a panel of 9 miRNAs for LUSC diagnosis. MiR-135b-5p, miR-196a-5p and miR-31-5p (LUAD) were found to be independent prognostic markers for survival in LUAD and miR-205 for LUSC (163).

From a practical perspective, the detection of miRNAs in liquid biopsy is constantly evolving and perfecting, as miRNAs are the most studied ncRNAs in liquid biopsies (164). According to Ma et al., several detection methods have been used for miRNAs, including qPCR, rolling circle amplification, strand displacement amplification and hybridization chain reaction (164). Although the detection of miRNAs in liquid biopsy holds great potential for future, current large-scale use is hampered by numerous factors. Limitations in moving from preclinical models to clinical applications include the need for validation in large-scale population (165), laboratory standardization (164), differences in sample processing techniques (166), and others.

The reproducibility of these markers/panel of miRNAs in clinical practice is seldom with great success. A major limitation is the reproducibility in independent cohorts due to variability in several parameters, including patient characteristics (e.g., age, sex), sample collection, analytical miRNA detection strategies, and others. Furthermore, miRNA expression is modulated via multiple methods, such as RNA-binding proteins (167), marking their potential variability between subjects. A future direction is also represented by the integration of miRNA panels with genomic and epigenomic data, leading to a more comprehensive characterization of the tumor. Yang et al. showed that there is a crosstalk between miRNA expression for immune regulation, DNA methylation and copy number variation in glioma (168).

When considering miRNAs in a therapeutic context, the agents can act either as miRNA mimics or miRNA inhibitors. **Table 4** presents a selection of NSCLC preclinical studies that focus on miRNA mimics.

**Table 4:** Examples of miRNA mimics in NSCLC preclinical studies.

MiRNA mimic	Preclinical model/*in vivo* model	Target	Delivery system	Effect	Ref
miR-34a	NSCLC tumor xenograft	c-Met, Bcl-2, partial repression of CDK4	Lipid-based delivery reagent	MiR-34a downregulates CDK4, c-Met and Bcl-2 in H460 cells and has tumor suppressive effects.	(169)
let-7b miR-34a	KRAS^G12D^ autochthonous NSCLC mouse model	Common targets: CDK6, MYC Other targets	Lipid-based delivery vehicle - NLE	In a KRAS-activated NSCLC mice model, the systemic delivery of let-7b/miR-34a mimics reduced lung tumor burden.	(170)
miR-200c	NSCLC tumor xenograft	PRDX2 GABP/Nrf2 SESN1	Liposomal nanoparticle NOV340/miR-200c	MiR-200c increased intracellular ROS levels and p21 levels.	(171)
5-Fluorouracil-miR-129 (5-FU-miR-129)	NSCLC metastasis model	Bcl-2 HMGB1	Vehicle-free delivery	5-FU-miR-129 inhibited cancer cell proliferation and induced apoptosis in A549/Calu-1 cell lines. This modified miRNA overcame the *in vitro* resistance to erlotinib or 5-FU. When referring to the *in vivo* effects, 5-FU-miR-129 inhibited tumor growth and led to an increase in survival.	(172)

## 5 Conclusion

In the present paper, we have critically reviewed the main prospects pertaining to the effects of miRNAs in the personalized medicine of lung cancer.

Several mutated/genetically altered genes drive lung cancer to a more aggressive phenotype. For instance, *EGFR L858R* point mutation in exon 21 and the LREA in-frame deletion on exon 19 are two *EGFR* activating mutations, which are druggable via *EGFR* TKIs. *ALK* rearrangements lead to constitutive *ALK* kinase activity, with lung tumors exhibiting increased aggressive behavior. This includes nodal metastasis and advanced stages at diagnosis; these *ALK* rearrangements are also druggable via targeted therapeutics such as crizotinib. Furthermore, lung cancer tumors harboring *KRAS* mutations carry usually a poor prognosis; *KRAS* activation induces interactions with several key pathways, such as the *RAF/MEK/ERK* pathway and the *PI3K/AKT* pathway. Targeted therapeutics are also available for patients harboring *KRAS* mutation - sotorasib, or adagrasib for *KRAS* G12C mutation. Other well-known genetic alterations that may be encountered in lung cancers include *ROS1* rearrangements, *RET* translocations, alterations in the *MET* gene, *NTRK* fusions and *BRAF* mutations. Herein, we also provide a selection of miRNAs that target these essential driver genes/signaling pathways in lung cancer - for instance, miR-33a and miR-128b that target the *EGFR* pathway, miR-200c and miR-449 that target the *ALK* signaling, miR-205 that interferes with *MET*, miR-148a-3p that is tumor suppressive on the *RAS/MAPK/ERK* signaling and miR-31 that has pro-oncogenic effect by modulating the *RAS/MAPK* pathway.

Furthermore, for a better integrative overview, our paper critically discusses the main cancer processes that govern lung cancer progression, along with essential prospects about miRNA modulation of these processes. We underline the implication of miR-28 as a promoter of cancer cell proliferation and miR-218 as a suppressor of invasion and metastatic spread via targeting *Slug*, *ZEB2* and EMT. MiR-29c acts as a tumor suppressor via targeting *VEGFA* and several miRNAs have been described to be implicated in therapy resistance - miR-378, miR-328, miR-589, miR-1244.

When considering potential clinical applications and future directions in miRNA research in lung cancer, our paper highlights several developments in the field of lung cancer biomarkers - a combined peripheral blood-derived miR-200 EVs with CA242, CEA and CA199 that displays high diagnostic performance in early-stage NSCLC and also other miRNA diagnostic panels that may prove useful in early diagnosis or even in discriminating LUAD from LUSC. Although the liquid biopsy strategy is not entirely standardized and developed, it appears that the constant evolution of the field holds great potential.

The field of lung cancer is rapidly advancing, driven by the high mortality rates associated with this malignancy. The addition of ncRNAs and specifically of miRNAs into the complex molecular framework of lung cancer has the potential to improve patient care. Although there are still numerous aspects that need to be apprehended for a better integration of miRNA study in lung cancer, significant steps forward have already been achieved, as highlighted in this paper.
